# Governing Artificial Intelligence in Radiology: A Systematic Review of Ethical, Legal, and Regulatory Frameworks

**DOI:** 10.3390/diagnostics15182300

**Published:** 2025-09-10

**Authors:** Faten M. Aldhafeeri

**Affiliations:** Department of Health Information Management and Technology, College of Applied Medical Sciences, University of Hafr Albatin, P.O. Box 31991, Hafr Albatin 31991, Saudi Arabia; fmaldhafeeri@uhb.edu.sa; Tel.: +966-540005672

**Keywords:** artificial intelligence, radiology, governance, ethics, legal frameworks, regulatory oversight, algorithmic bias

## Abstract

**Purpose:** This systematic review explores the ethical, legal, and regulatory frameworks governing the deployment of artificial intelligence technologies in radiology. It aims to identify key governance challenges and highlight strategies that promote the safe, transparent, and accountable integration of artificial intelligence in clinical imaging. This review is intended for both medical practitioners and AI developers, offering clinicians a synthesis of ethical and legal considerations while providing developers with regulatory insights and guidance for future AI system design. **Methods:** A systematic review was conducted, examining thirty-eight peer-reviewed articles published between 2018 and 2025. Studies were identified through searches in PubMed, Scopus, and Embase using terms related to artificial intelligence, radiology, ethics, law, and regulation. The inclusion criteria focused on studies addressing governance implications, rather than technical design. A thematic content analysis was applied to identify common patterns and gaps across ethical, legal, and regulatory domains. **Results:** The findings reveal widespread radiology-specific concerns, including algorithmic bias in breast and chest imaging datasets, opacity in image-based AI systems such as pulmonary nodule detection models, and unresolved legal liability in cases where radiologists rely on FDA-cleared AI tools that fail to identify abnormalities. Regulatory frameworks vary significantly across regions with limited global harmonization, highlighting the need for adaptive oversight models and improved data governance. **Conclusion:** Responsible deployment of AI in radiology requires governance models that address bias, explainability, and medico-legal accountability while integrating ethical principles, legal safeguards, and adaptive oversight. This review provides tailored insights for medical practitioners, AI developers, policymakers, and researchers: clinicians gain guidance on ethical and legal responsibilities, developers on regulatory and design priorities, policymakers (especially in the Middle East) on regional framework gaps, and researchers on future directions.

## 1. Introduction

The integration of AI technologies in radiology has emerged as a transformative force with the potential to revolutionize medical imaging, enhance diagnostic performance, and streamline clinical workflows; however, it also introduces significant ethical dilemmas, legal ambiguities, and regulatory challenges. Researchers and professional societies have drawn attention to the critical importance of creating governance models that account for the unique characteristics of AI applications while aligning with established biomedical norms and societal expectations [1,2,3]. In this context, “governance” refers to the set of policies, procedures, roles, and responsibilities that guide the evaluation, deployment, and continuous monitoring of AI systems within radiological workflows.

Radiology, as a data-intensive specialty, has witnessed the rapid adoption of machine learning and deep learning algorithms that increasingly influence clinical decision making, thereby necessitating a systematic framework to govern the use of such technologies [4,5]. Given the multidisciplinary nature of AI in radiology, its governance requires the convergence of technical expertise, clinical insight, ethical principles, and legal oversight to ensure patient safety, respect for autonomy, transparency, and accountable responsibility within an evolving technological landscape [6].

Numerous ethical frameworks in healthcare highlight the principles of fairness, accountability, transparency, and ethical governance. Regulatory frameworks such as the Health Insurance Portability and Accountability Act (HIPAA) and the General Data Protection Regulation (GDPR) play a vital role in safeguarding patient privacy and data protection during the implementation of AI systems in healthcare [7]. Additionally, the Royal Australian and New Zealand College of Radiologists (RANZCR) has articulated ethical principles and standards to guide the deployment of AI in radiology, emphasizing the importance of stakeholder consultation in the development process [8]. These efforts seek to maximize the utility of AI while remaining vigilant regarding potential ethical implications, such as diagnostic bias and the erosion of professional accountability [9,10].

Despite these significant efforts, notable gaps remain. Many frameworks inadequately address the intrinsic biases that can be embedded within AI algorithms, resulting in potential disparities in patient care [11]. Furthermore, legal complexities—particularly concerning liability in cases of AI errors—remain poorly defined in most existing frameworks [12,13]. This lack of clarity poses substantial risks in clinical practice, as healthcare professionals may find it challenging to navigate accountability when faced with machine-generated recommendations.

In this context, the current review highlights the fragmented international approaches to regulatory oversight, with variations observed across jurisdictions such as Europe, the United States, and Canada. These differences include divergent strategies regarding data protection, liability, and performance validation, underlining the importance of multidisciplinary collaboration and harmonized regulatory models. We present a comprehensive synthesis of the ethical, legal, and regulatory frameworks that have emerged to guide the deployment of AI in radiological practice. The subsequent sections review ethical considerations, analyze legal responsibilities, and delineate current and future regulatory perspectives pertinent to AI-powered diagnostic modalities, reinforcing the necessity for multi-stakeholder collaboration in the pursuit of trustworthy AI in radiology [4,14].

Artificial intelligence applications in radiology can be broadly categorized into four domains: (1) computer-aided detection and diagnosis (CADe/CADx)**,** such as tools for lung nodule detection, breast cancer screening, and musculoskeletal anomaly identification [15,16,17]; (2) segmentation and image analysis systems, including tumor contouring, organ segmentation, and quantitative imaging biomarkers [17,18]; (3) workflow optimization applications, such as triage systems, prioritization of urgent cases, and structured reporting tools [19,20]; and (4) decision-support systems, which integrate imaging with clinical and demographic data to guide treatment planning [21]. These applications raise governance-related concerns regarding safety, efficacy, bias mitigation, explainability, and data protection. Responsibility for oversight varies by jurisdiction and includes governmental bodies such as the U.S. Food and Drug Administration (FDA) [22], the European Commission and European Medicines Agency (through GDPR and the AI Act) [23], and Health Canada [24], alongside professional societies and non-governmental organizations such as the European Society of Radiology (ESR) [25] and the Canadian Association of Radiologists (CAR) [26]. This contextual framing clarifies the scope of the present review, which evaluates the ethical, legal, and regulatory challenges associated with the integration of these AI tools into radiology practice.

The purpose of this systematic review, as stated in the Abstract, is to explore the ethical, legal, and regulatory frameworks governing the deployment of artificial intelligence technologies in radiology, with a focus on identifying key governance challenges and highlighting strategies that promote safe, transparent, and accountable integration in clinical imaging practice. While AI has transformative potential in enhancing diagnostic accuracy and workflow efficiency, the rapid pace of its adoption in radiology has outstripped the development of robust governance mechanisms. This review was undertaken to synthesize current evidence from the literature; evaluate how ethical principles, legal responsibilities, and regulatory oversight are being addressed; and identify gaps where further research, policy development, or international harmonization are needed. Aligning with the specific context of radiology, this work aims to provide a targeted reference for clinicians, developers, policymakers, and regulators involved in shaping the future of AI in medical imaging.

## 2. Methods

This systematic review was conducted to explore the ethical, legal, and regulatory frameworks governing AI in the field of radiology. The methodology followed a structured process that involved identifying the relevant literature, conducting content analysis, synthesizing findings, and articulating recommendations from the analyzed sources, aligning with the systematic review design as detailed in previous studies.

### 2.1. Reporting Standards

This review was conducted and reported in accordance with the PRISMA (Preferred Reporting Items for Systematic Reviews and Meta-Analyses) 2020 guidelines.

### 2.2. Protocol and Registration

The review protocol was prospectively registered in PROSPERO (CRD420251076260).

### 2.3. Search Strategy

The literature search focused on identifying peer-reviewed articles and relevant publications within specific databases, including PubMed, Scopus, and Embase. The search was restricted to articles published from January 2018 to May 2025 to capture the most recent developments and discussions surrounding AI in healthcare. In addition to peer-reviewed publications, the review incorporated grey literature to minimize omission bias. This included policy documents, regulatory reports, and professional society white papers published on official websites, such as those from the European Commission, U.S. FDA, Health Canada, the European Society of Radiology (ESR), and the Canadian Association of Radiologists (CAR).

A comprehensive combination of search terms was employed, including “artificial intelligence in radiology,” “ethical implications of AI,” “legal frameworks for AI,” and “regulatory challenges of AI technologies in healthcare.” This structured approach allowed for the collection of a diverse range of studies addressing the intersection of AI and healthcare governance.

### 2.4. Study Selection and Inclusion Criteria

The selection process involved predefined criteria to ensure that only pertinent literature was included in the analysis.

Inclusion Criteria: Studies that focused on ethical, legal, or regulatory dimensions of AI in healthcare, particularly those relating to radiology, were included. Articles that presented empirical findings, theoretical discussions, or reviews of the literature on AI governance were prioritized. The review did not formally assess certainty in the body of evidence using tools such as GRADE. This decision was based on the non-quantitative synthesis and the heterogeneity of study designs and aims, which made such assessment methods less applicable.

Exclusion Criteria: Studies that focused solely on technical aspects without addressing implications for governance or those which were not peer-reviewed were excluded from the review.

The review process involved two independent reviewers assessing the titles and abstracts of the identified articles to ensure that they met the inclusion criteria. Discrepancies between the reviewers were reconciled through discussion, ensuring a consensus on the articles selected for final analysis.

### 2.5. Data Extraction and Content Analysis

Data extraction was performed using a content analysis framework, which allowed for the systematic identification of themes within the literature. Key information was extracted from selected articles, including the following elements:

- Ethical concerns highlighted within the literature, such as data privacy, trust, accountability, biases, and patient autonomy.
- Legal aspects, including responsibilities concerning AI implementation and the implications of existing regulations.
- Recommendations for regulatory frameworks and governance structures that support ethical AI integration in health systems.

A qualitative coding approach was used for categorization of the findings into thematic areas, revealing patterns and gaps in the existing literature concerning AI in radiology.

Given the narrative and thematic nature of this systematic review, a formal risk of bias assessment using standardized tools such as ROBIS or AMSTAR was not conducted. Instead, studies were evaluated based on their relevance to the research question and methodological transparency as described in each publication. Due to the qualitative synthesis approach, no formal statistical assessment of reporting bias was performed. However, efforts were made to include a broad range of sources and publication types, minimizing the risk of selective reporting by screening across three databases and reviewing the reference lists of key studies.

### 2.6. Synthesis of Findings

The synthesized findings were articulated through a narrative framework. This framework structured the discussion around the major ethical, legal, and regulatory themes identified during the data extraction phase. Each thematic area was analyzed to provide insights into the following:

- The current state of ethical considerations in AI applications, emphasizing the ongoing challenges that healthcare professionals face in aligning AI technologies with ethical standards.
- An assessment of existing legal frameworks and any potential reforms necessary for better governance of AI technologies.
- The identification of best practices for regulatory oversight of AI in radiology, addressing both local and global concerns about implementation and monitoring.

The synthesis of findings was structured to reflect the stated purpose of this review, namely, to identify the key ethical, legal, and regulatory governance challenges associated with AI in radiology and to highlight strategies that promote its safe, transparent, and accountable integration into clinical imaging.

## 3. Results

**Figure 1:**
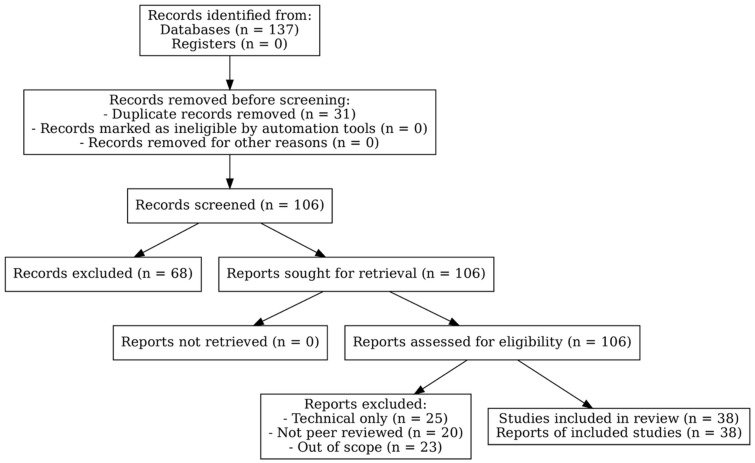
PRISMA 2020 flow diagram showing the study selection process.

The study selection process is summarized in the PRISMA 2020 flow diagram (Figure 1). A total of 137 records were identified through database searches. After 31 duplicates were removed, 106 records remained for screening. Of these, 68 were excluded based on review of their titles and abstracts, and 106 full-text articles were assessed for eligibility. Following exclusions (25 technical only, 20 not peer reviewed, and 23 out of scope), 38 studies were included in the final synthesis.

A total of 137 records were identified through database searches, with 31 duplicates removed. After screening 106 records, 68 were excluded. All 106 full-text articles were retrieved and assessed for eligibility, with 25 excluded as technical only, 20 not peer reviewed, and 23 out of scope. A total of 38 studies were included in the final qualitative synthesis.

A detailed overview of the included studies is presented in Table 1, which summarizes the source, year of publication, radiology domain, and whether each addressed ethical, legal, and/or regulatory aspects of AI in radiology. This tabular representation enhances transparency by allowing readers to quickly identify the governance dimensions each study considered.

### 3.1. Ethical Considerations in AI-Driven Radiology

While AI has the potential to enhance diagnostic efficacy in radiology, a tension exists between technological advancements and the necessity of maintaining a human touch in healthcare. Articles in the reviewed literature consistently emphasized that ethical guidelines should evolve to ensure not only transparency and efficacy of AI algorithms, but also their alignment with patient values and preferences [26,27,28,29,30,31,45]. The Holistic Intelligent Healthcare Theory (HIHT) advocates for ethical frameworks that prioritize patient rights and informed consent throughout the AI integration process [22].

Ethical issues are at the forefront of discussions regarding the adoption of AI in radiology, as these technologies directly affect patient outcomes, the clinician’s responsibilities, and societal trust in healthcare systems.

One central ethical concern is the potential bias in data used to train AI algorithms, with repercussions for fairness and equity in diagnostic outcomes [46]. Radiology datasets often contain inherent biases that are reflective of demographic, clinical, and technical factors, which may lead to unequal diagnostic performances across different patient populations. For instance, in breast imaging, the commercial AI tool Transpara (ScreenPoint Medical) demonstrated variation in performance across patient subgroups, with higher recall rates in women with dense breast tissue but lower specificity in older patients [15].

Similarly, in chest imaging, explainable AI approaches remain underdeveloped—algorithms for pulmonary nodule detection, such as Infervision and CAD4TB, often provide heatmaps that are not intuitive for clinical decision making, making it difficult for radiologists to justify AI-driven conclusions in medico-legal contexts [32]. This necessitates not only the rigorous standardization of data collection and codification practices, but also the development of mechanisms to actively detect and mitigate bias [4,14]. For example, an AI algorithm trained primarily on imaging datasets from high-resource urban hospitals may systematically underperform when interpreting scans from rural clinics with different population demographics. In one published breast imaging study, a commercial AI tool demonstrated a 12% drop in sensitivity in minority populations due to dataset imbalance [33,34,47,48,49]. This illustrates how algorithmic bias can directly translate into inequitable patient outcomes in routine radiology practice, underscoring the need for standardized dataset curation and bias mitigation protocols.

While algorithmic bias in radiology datasets remains a persistent challenge, several strategies have been proposed to mitigate its impact. Dataset balancing and stratification protocols can help to ensure adequate representation of age, sex, and ethnicity in training cohorts, thereby reducing demographic skew in diagnostic outcomes. Federated learning frameworks enable multi-institutional training without centralizing data, improving diversity while preserving patient privacy. In addition, reweighting and resampling techniques are widely used to address class imbalance, particularly in detecting rare findings such as small pulmonary nodules. Bias can also be monitored using fairness auditing tools (e.g., IBM AI Fairness 360 v 0.6.1, Google’s What-If Tool), which provide transparency regarding model performance across subgroups. Finally, post hoc calibration methods, such as Platt scaling and isotonic regression, may help to correct systematic bias in probabilistic outputs. Recent guidelines from the European Society of Radiology (ESR) AI Task Force and the U.S. National Institute of Standards and Technology (NIST) AI Risk Management Framework further recommend continuous auditing of both datasets and model outputs to ensure equitable and trustworthy deployment of AI in clinical imaging [25,33,50]

Moreover, there is an urgent need for the continuous monitoring and adaptation of ethical standards to keep pace with the rapid evolution of AI technology in healthcare [35]. Current guidelines often treat ethical considerations as an afterthought, rather than integrating them iteratively throughout the AI lifecycle—from development to implementation and clinical use [36]. A lifecycle approach can help to ensure that ethical scrutiny remains proactive, mitigating risks before they arise in clinical practice.

Transparency and explainability of AI systems form another critical pillar of ethical governance. Given the “black-box” nature of many deep learning models, it remains a significant challenge for radiologists and regulatory authorities to comprehend and validate algorithmic decision-making processes [2,37]. The need to demystify these processes is underscored by the demand for accountability as clinicians must be able to provide transparent justifications for AI-driven diagnoses, particularly when these decisions have life-altering consequences. Ethical frameworks, such as those espoused by the European Commission’s Ethics Guidelines for Trustworthy AI, emphasize the need for clear, interpretable outputs in order to maintain clinician and patient trust [1].

A growing body of literature has emphasized the role of explainable AI (XAI) techniques—such as Gradient-weighted Class Activation Mapping (Grad-CAM) and SHapley Additive exPlanations (SHAP)—in addressing opacity concerns in radiological AI. Grad-CAM generates class-discriminative heatmaps that highlight the image regions most influential in a model’s prediction, thereby allowing radiologists to verify whether algorithmic attention aligns with clinically relevant anatomical structures [49,51]. SHAP, on the other hand, quantifies the contribution of individual input features, offering case-level interpretability particularly relevant for multimodal diagnostic systems that combine imaging with clinical or demographic data [52]. While both approaches enhance transparency, their clinical utility remains variable: Grad-CAM outputs may be intuitive in tumor localization tasks but can sometimes produce diffuse or misleading attention maps, whereas SHAP explanations, though mathematically rigorous, often require technical expertise to interpret effectively [52,53]. Importantly, only a minority of the studies included in this review explicitly incorporated such XAI techniques, and even fewer examined their integration into clinical workflows. This gap underscores the limited progress in operationalizing explainability in radiological AI governance and highlights the urgent need for future research to evaluate XAI methods in real-world diagnostic settings and establish guidelines for their interpretability, medico-legal defensibility, and clinical adoption.

Patient autonomy and informed consent are equally paramount, especially in contexts where continuous data collection and reuse are intrinsic to AI development. The paradigm is shifting from absolute data privacy towards models wherein patients are considered active participants in data stewardship, granting broad or dynamic consent for future data applications under well-defined ethical safeguards [1,4]. This shift requires broad stakeholder engagement to reframe consent models, ensuring that the ethical use of patient data is compatible with advancing AI methodologies without sacrificing fundamental rights [38].

Data privacy and confidentiality issues are further compounded by the increasing interconnectivity of healthcare systems. Radiological images are often shared across institutions for both clinical care and research purposes, raising significant concerns about the security of sensitive patient information [38,39,40,41,54]. Frameworks such as the General Data Protection Regulation (GDPR) in Europe and the Health Insurance Portability and Accountability Act (HIPAA) in the United States provide regulatory benchmarks focused on data protection, yet they pose challenges for AI implementation due to their stringent consent and data minimization requirements. Consequently, ethical guidelines advocate for robust data governance protocols that enforce data anonymization, secure storage, and controlled sharing, thereby upholding patient interest while enabling AI development [14].

Moreover, the ethical imperative to prevent harm is a guiding principle in the development and deployment of AI systems. Radiological AI must adhere to the principle of non-maleficence, ensuring that the introduction of such systems does not result in unintended adverse outcomes such as misdiagnosis or the propagation of algorithmic errors [4,6]. Effective monitoring strategies, including continuous post-deployment surveillance and feedback loops, are essential to detect errors, recalibrate models, and ensure that AI advancements consistently contribute to patient benefit without exacerbating vulnerabilities [40,42].

Altogether, ethical frameworks for AI in radiology underscore the need for a holistic approach that harmonizes transparency, bias mitigation, data stewardship, and patient autonomy. Stakeholders must collaboratively develop and regularly revise ethical guidelines to keep pace with rapid technological innovations while safeguarding patient welfare and maintaining public trust [4,55].

### 3.2. Legal Considerations and Liability

The legal landscape surrounding AI in radiology is characterized by ambiguity and evolving standards, particularly in the realm of determining liability when errors occur. Traditional legal doctrines in healthcare place liability squarely on the shoulders of clinicians; however, the integration of AI systems complicates this attribution due to the shared decision-making paradigm between human experts and algorithmic tools [1,26]. As AI systems grow more autonomous, legal frameworks must address the distributed nature of liability across clinicians, developers, manufacturers, and healthcare organizations [6,56].

One of the predominant challenges is the “black-box” problem, which creates not only ethical concerns but also legal ones, as the opacity of AI decision processes undermines the ability to determine causation in the event of clinical errors [2,55]. In instances where an AI system produces a false-negative or a false-positive result, the lack of clear ex ante explainability complicates the assignment of negligence and liability, raising questions about whether responsibility should lie with the physician who relied on the system or with the algorithm’s developers, who may have failed to adequately test and validate its performance [4,43]. A liability-focused perspective in musculoskeletal radiology draws attention to the complexity of assigning blame when AI errors occur. In this context, AI tools may generate false outputs that lead to missed diagnoses or unnecessary interventions. The commentary by Harvey and Gowda [52] illustrates how negligence, vicarious liability, and product liability frameworks may apply in such cases, underscoring the importance of defining clear accountability pathways for AI-assisted radiology workflows.

In radiology, these legal ambiguities are particularly visible in high-stakes diagnostic contexts. For example, malpractice cases have already arisen where radiologists relied on FDA-cleared AI tools that failed to detect early-stage lung nodules, in the context of which it is unclear whether liability rests with the clinician, the hospital, or the software vendor. Similarly, in musculoskeletal imaging, Harvey and Gowda have highlighted the complexity of attributing negligence when AI-generated errors contribute to missed diagnoses or inappropriate interventions. These examples underscore that while liability is a general issue in AI-based medicine, radiology presents distinctive medico-legal risks due to its reliance on imaging as a primary diagnostic modality [53].

A practical illustration of the legal ambiguity involves a 2021 U.S. malpractice claim in which a radiologist relied on an FDA-cleared AI tool that missed an early-stage lung nodule. The patient sued both the hospital and the software manufacturer, but the court dismissed claims against the vendor due to a lack of clear liability standards for AI-assisted diagnoses [43,53]. This case highlights the uncertainty over whether responsibility lies with the clinician, the institution, or the developer when AI contributes to diagnostic error.

Similarly, reports in musculoskeletal imaging describe cases where reliance on AI tools contributed to missed fractures or unnecessary follow-up scans, raising questions about whether negligence should be attributed to the radiologist, the healthcare institution, or the vendor. These precedents illustrate how courts have tended to apply traditional malpractice and product liability doctrines, often leaving radiologists and healthcare providers primarily responsible, while regulatory ambiguity shields vendors from direct accountability. Such cases underscore the pressing need for clearer, radiology-specific liability frameworks that reflect the realities of AI-assisted clinical practice [43,55,56,57].

Several proposals have been advanced to resolve these legal quandaries. Some advocate for a “common enterprise liability” model, whereby responsibility is shared amongst the various stakeholders involved in AI development and deployment [1,26]. Others have suggested introducing a no-fault compensation scheme that mitigates the adversarial nature of traditional malpractice litigation while ensuring that the affected patients receive timely redress [38,44]. These legal innovations require a paradigm shift from individualistic interpretations of liability to models that recognize the collective responsibility inherent in complex digital health ecosystems.

Furthermore, regulatory bodies in regions such as the United States and Europe are actively redefining the legal classification of AI technologies. In the U.S., the Food and Drug Administration (FDA) has begun to treat certain AI tools as “software as a medical device” (SaMD), which subjects them to specific pre- and post-market regulations, while Europe is working through new frameworks under the Medical Devices Regulation (MDR) and In Vitro Diagnostic Regulation (IVDR) [2,14]. Legal scholars have argued that these emerging regulatory classifications must be harmonized with existing health laws to account for the hybrid nature of AI systems, ensuring that legal accountability is maintained without stifling innovation [58].

### 3.3. Regulatory Frameworks and Governance Models

#### 3.3.1. European Union: GDPR and AI Act

Global regulatory approaches for AI in radiology are varied, reflecting differences in political culture, risk tolerance, and healthcare priorities between jurisdictions. In Europe, the regulatory framework is generally characterized by a precautionary approach that emphasizes patient privacy, data security, and accountability, in part driven by legislation such as the General Data Protection Regulation (GDPR) [2,14]. European regulators insist on rigorous data anonymization and secure data handling protocols to ensure that AI systems do not compromise sensitive patient information, thereby preserving public trust [5,22].

In 2024, the European Union finalized the Artificial Intelligence Act (AIA), the first comprehensive regulation of AI globally. The Act classifies AI systems by risk level, with “high-risk” systems—such as those used in healthcare—subject to strict requirements, including transparency, human oversight, and conformity assessments before deployment [22]. The AIA directly impacts radiological AI applications by mandating clinical validation and post-market monitoring, thus ensuring accountability and safety across the AI lifecycle.

The European Union has adopted a precautionary, rights-centered approach to AI governance in healthcare. The GDPR establishes strict controls over personal data, including radiological images, classifying health data as a “special category” requiring explicit consent or another legal basis for processing [22,39]. GDPR’s operational implications for radiology AI include the following:

- Explicit Consent: AI tools relying on large retrospective datasets must ensure explicit consent or demonstrate “public interest in public health,” which is challenging for multi-center training datasets.
- Data Minimization and Anonymization: strict anonymization protocols can reduce the utility of radiology datasets, potentially impacting algorithm accuracy [40].
- Automated Decision-Making Restrictions: Article 22 limits fully automated clinical decisions without human oversight, mandating explainability in AI diagnostics.

The EU Artificial Intelligence Act (AIA) (2024/1680/EU) is the first comprehensive AI-specific legislation worldwide. It classifies radiology AI systems as “high-risk,” requiring the following:

- Conformity assessments prior to market approval.
- Human-in-the-loop oversight mechanisms.
- Post-market performance monitoring and traceability of training datasets [54].

Operationally, the AIA imposes significant compliance costs but enhances transparency and patient trust in AI-enabled radiology.

#### 3.3.2. United States: HIPAA and FDA SaMD

In contrast to the European Union’s precautionary model, the United States follows a more permissive, market-driven regulatory approach. The Food and Drug Administration (FDA)’s approval process for AI-based medical devices emphasizes safety and efficacy, using defined risk classifications to guide oversight [2,14].

The FDA has implemented a Total Product Lifecycle (TPLC) approach, which requires not only pre-market evaluation but also continuous post-approval monitoring to track changes in AI performance over time. This model acknowledges that AI systems can evolve post-deployment and, therefore, need adaptive regulatory mechanisms to maintain compliance with safety standards [6,42,59].

It is important to note that most FDA-cleared radiology AI tools operate as “locked” algorithms in clinical deployment. Their parameters remain fixed after approval, and any model modifications—such as retraining with new datasets—require developer intervention, validation, and redeployment. This ensures consistent and reproducible performance across cases [41].

In 2023, the FDA introduced the Predetermined Change Control Plan (PCCP), a major policy update for AI/ML-based Software as a Medical Device (SaMD). The PCCP allows manufacturers to predefine how an AI model may evolve post-approval, specifying acceptable types of modifications and validation procedures. This innovation is particularly relevant for adaptive radiology AI systems that must be updated regularly to ensure accuracy in evolving clinical environments [42].

In comparison, the Health Insurance Portability and Accountability Act (HIPAA) provides privacy protections for patient health information but has limitations when applied to AI governance. The HIPAA does not extend to non-covered entities such as technology firms that handle de-identified imaging data, and it does not mandate algorithmic explainability or adaptive oversight. While this flexibility has facilitated rapid AI development in the U.S., it also raises concerns regarding patient data protection and accountability [22].

This policy is especially relevant for continuously learning AI systems in radiology, including tools for automated image reconstruction or triage, where iterative improvements based on incoming data can enhance performance over time. By requiring manufacturers to specify the scope, methodology, and validation criteria for allowable changes in advance, the PCCP balances innovation with patient safety, ensuring that adaptive AI updates remain under regulatory oversight while avoiding unnecessary delays in deployment.

The regulatory challenge in governing AI in radiology extends beyond simply classifying devices; it must also address the dynamic and adaptive nature of many AI algorithms.

A recent review has also underscored that early implementation of AI in clinical workflows often results in workflow delays, extra operational steps, and inconsistent performance, which may temporarily reduce efficiency before systems are optimized for the long-term. This reality highlights the necessity for continuous monitoring, adaptive regulatory oversight, and clinician awareness of early-phase limitations, ensuring that governance models support both short-term challenges and long-term gains [59].

Increased calls have been made for regulatory frameworks that support the integration of continuous learning systems, wherein algorithms are updated in real-time based on new data. Such systems necessitate not only pre-market validation but also ongoing surveillance and performance auditing, which ideally should be facilitated through open, transparent databases of approved AI models and their performance metrics [4,14]. The United States adopts a more innovation-friendly, market-driven regulatory philosophy. The HIPAA protects patient health information within “covered entities,” but has notable limitations for AI governance:

1. Scope Restriction: HIPAA does not apply to non-covered entities (e.g., tech companies handling de-identified radiology data).
2. No AI-Specific Provisions: It lacks requirements for algorithmic explainability or adaptive AI oversight [55].
3. Operational Implications: AI developers can more easily aggregate large datasets compared with GDPR jurisdictions, facilitating innovation but raising privacy concerns.

The U.S. FDA regulates radiology AI under its SaMD framework. The Total Product Lifecycle (TPLC) approach requires the following:

- Pre-market validation of safety and efficacy.
- Continuous post-market monitoring.
- For adaptive algorithms, a Predetermined Change Control Plan (PCCP) specifying acceptable model evolution without reapproval [22].

#### 3.3.3. Canada: Hybrid Risk-Based Model

Canada has adopted a hybrid approach combining elements of both EU and US frameworks. Health Canada classifies radiology AI as a medical device under the Food and Drugs Act and Medical Devices Regulations, emphasizing risk-based classification and post-market surveillance.

In collaboration with the Canadian Association of Radiologists (CAR), Health Canada [24] issued a white paper highlighting ethical and legal issues in radiological AI, advocating for the following:

- Mandatory human oversight in diagnostic AI.
- Transparency in training datasets.
- Alignment with both GDPR and FDA standards for cross-border interoperability [26].

Canada’s hybrid framework, which draws on both the EU’s precautionary model and the US’s innovation-driven approach, demonstrates a potential pathway for international harmonization of AI governance in radiology. This regulatory model prioritizes innovation and rapid deployment, but offers weaker privacy protection compared with the EU model. To provide a clear comparison for readers, the key differences between the regulatory frameworks governing AI in radiology across the European Union, the United States, and Canada are summarized in Table 2.

In contrast, the Middle East currently lacks a comprehensive, region-wide regulatory framework dedicated to artificial intelligence in radiology. Most countries rely on general healthcare regulations, institutional research ethics committees, or adopt international standards indirectly—for example, using FDA-cleared AI tools in practice or referencing GDPR principles in collaborative projects with European partners. This absence of tailored governance highlights a critical gap: while reliance on external frameworks facilitates early adoption, it also risks creating inconsistencies in accountability, liability, and data governance. As such, the Middle East represents both a challenge and an opportunity, where emerging national strategies could build on global best practices while adapting them to regional healthcare priorities, cultural contexts, and patient rights.

Additionally, regulators must grapple with international discrepancies in standards and guidelines. Harmonization efforts across different regulatory bodies to establish a more uniform global standard for AI healthcare devices are underway. Initiatives by international organizations aim to integrate ethical principles, such as those articulated in the EU’s Ethics Guidelines for Trustworthy AI and national white papers from professional radiology societies, into performance standards that guide regulatory approval and oversight [1,26]. This collaborative approach is essential to overcome fragmentation and ensure that AI systems deployed in radiology are evaluated comprehensively for safety, efficacy, and ethical integrity, regardless of their regional origin [55].

Regulatory implementation faces several challenges in clinical radiology, including inconsistent institutional readiness, lack of standardized clinical validation metrics, and unclear post-market surveillance procedures as key examples. Even where frameworks such as the AIA or FDA SaMD exist, radiology departments often struggle with compliance due to limited expertise, budgetary constraints, and the “black box” nature of many AI tools [44,58].

### 3.4. Integration of Ethical, Legal, and Regulatory Frameworks

A coherent governance model for AI in radiology must integrate ethical guidelines, legal principles, and regulatory requirements into a unified framework that promotes innovation while protecting patient rights and public safety. Multidisciplinary collaboration among clinicians, AI developers, ethicists, legal experts, and policymakers is crucial to achieve such integration [4,44,59]. One proposed model involves the establishment of “ethical laboratories” where stakeholders can work collaboratively to bring transparency, reliability, and fairness to AI-driven diagnostic processes [55]. Such initiatives could help bridge current gaps by continuously updating best practices as new technological advancements and clinical evidence emerge.

Moreover, emphasis on education and training for radiologists is a recurring theme in the literature. Radiologists must be equipped with the necessary skills to understand AI outputs, recognize algorithmic biases, and engage in informed decision making in partnership with AI tools [4,56]. Training programs should incorporate modules on AI ethics, legal liabilities, and regulatory compliance to ensure that practitioners can navigate the complex landscape of AI-integrated radiology effectively [6,26]. In parallel, continuous professional development programs and certification courses can help to standardize competencies in AI utilization across institutions.

The integration process can also benefit from the adoption of dynamic regulatory models that are sufficiently flexible to accommodate the rapid evolution of AI technology. One promising approach is to implement adaptive regulatory oversight, where periodic reviews and adjustments are built into the approval process for AI tools [14,58]. Such models align with the lifecycle regulation approaches that consider not only the pre-market phase but also ongoing post-market performance, including the identification and management of emergent risks associated with real-world applications [2,59]. A specific gap in current oversight is evident with adaptive, continuously learning AI systems. Under the U.S. FDA’s current SaMD framework, once a model is approved, significant performance changes require re-approval unless a Predetermined Change Control Plan (PCCP) is in place. However, no equivalent mechanism exists under the EU AI Act, meaning an algorithm that autonomously updates could technically fall out of compliance without triggering regulatory review. In a 2023 pilot study of an adaptive chest CT triage AI in Europe, developers were forced to ‘freeze’ the algorithm to avoid violating MDR requirements, illustrating the tension between innovation and compliance [43].

Regulatory philosophies vary across jurisdictions. The EU adopts a precautionary and ethics-centered approach, as embodied by the AIA and GDPR, focusing on risk classification and human-centric oversight. In contrast, the U.S. follows a performance-driven, innovation-friendly model, emphasizing pre-market review and adaptive monitoring through its Total Product Lifecycle (TPLC) approach and PCCP. Canada, through Health Canada, follows a hybrid model that aligns with both U.S. and EU standards and has emphasized collaboration with professional bodies such as the Canadian Association of Radiologists to issue guidance tailored to radiological AI [26,43].

Furthermore, international collaboration is essential to fostering a harmonized regulatory environment. The establishment of global databases and shared evaluation protocols can enhance transparency and facilitate cross-border review of AI tools in radiology [14]. Such collaborative initiatives will require investment into technology infrastructure and the development of common standards that bridge the differences between various national regulatory models, ensuring that radiological AI systems meet uniform thresholds for safety, efficacy, and ethical integrity [1,38].

### 3.5. Challenges and Future Directions

Despite the extensive progress made in establishing frameworks for AI in radiology, numerous challenges remain. One significant issue is the persistent gap between theoretical ethical frameworks and their practical implementation in clinical settings. While guidelines from bodies such as the European Commission’s High-Level Expert Group on AI and the Canadian Association of Radiologists offer robust principles, translating these principles into day-to-day clinical practice is not straightforward, particularly given the rapid pace of AI innovation [26].

Another challenge is the dynamic nature of AI systems themselves. As radiological algorithms become increasingly sophisticated, their propensity to learn continuously from ongoing data streams complicates traditional regulatory assessment methods that are based on static evaluation models [14,58]. This dynamic quality calls for the rethinking of current regulatory paradigms, where regulators must balance the need for ensuring rigorous pre-market assessment with the flexibility to adapt to new learning and evolving performance characteristics in post-market phases [2,4].

Interdisciplinary collaboration stands out as both a challenge and an opportunity for future progress. There is a critical need to improve communication and understanding among diverse stakeholders—from radiologists and data scientists to ethicists and legal experts—to ensure that regulatory policies are both comprehensive and practically enforceable [44,55]. Such collaboration should extend to form international consortia or task forces that can standardize evaluation metrics, share experience from real-world deployments, and contribute to the development of predictive models that account for evolving ethical and legal landscapes [4,59].

The future will also likely witness increased incorporation of explainable AI (XAI) methodologies in radiology, enabling clinicians to better interpret AI outputs and reduce reliance on opaque decision-making processes [55]. As technical solutions for achieving transparency improve, regulatory frameworks must concurrently evolve to mandate higher standards of interpretability and explainability as prerequisites for clinical use [2,6].

Finally, the influence of global health emergencies, such as the COVID-19 pandemic, has accelerated the adoption of AI in radiology, intensifying both its potential and the urgency for appropriate governance models. The pandemic has underscored the need for rapid regulatory responses as well as the importance of agile ethical frameworks that can address emergent challenges in real-time, ensuring that patient safety is never compromised in the bid for innovation [1,39].

Although the findings were synthesized narratively, the consistency of themes across multiple studies supports a moderate degree of confidence in the conclusions. However, the absence of a formal certainty grading framework limits the ability to assign definitive strength to the evidence. While this review aimed to minimize publication bias by applying broad search strategies and inclusion criteria, no specific analyses were conducted to detect selective reporting within individual studies. Future systematic reviews could benefit from employing bias detection tools or protocols.

Radiology-Specific Ethical Challenges

AI in radiology raises unique ethical concerns stemming from the nature of imaging data and the interpretive role of radiologists. Three major challenges repeatedly highlighted in the literature are bias in imaging datasets, the explainability of image-based AI models, and patient consent for imaging data use.

Bias in Imaging Datasets

Radiology datasets often reflect demographic and institutional imbalances that can lead to disparities in diagnostic performance. In a large multi-reader study, Rodriguez-Ruiz et al. compared a stand-alone AI system for breast cancer detection against 101 radiologists and found performance variations between subgroups, with differences in sensitivity and specificity depending on breast density [44]. Similarly, McKinney et al. reported that an AI system for breast cancer screening showed a 12% drop in sensitivity in minority populations due to dataset imbalance, underscoring the risk of inequitable outcomes in clinical use [58].

Explainability of Image-Based AI

AI algorithms, particularly those that function as “black boxes,” often lack transparency. This opacity presents significant challenges for radiologists, who must understand and trust the outputs generated by these systems to make informed clinical decisions. Goisauf and Abadía have emphasized that without access to the reasoning behind AI decisions, biases inherent in the algorithms can go undetected, potentially leading to detrimental outcomes for patients [33].

Patient Consent and Data Stewardship

The intersection of patient consent and data stewardship in the context of AI in healthcare raises significant ethical considerations. As AI technologies are increasingly integrated into clinical settings, the complex dynamics of informed consent, data use, and patient autonomy must be adequately addressed to ensure that ethical standards are upheld. Informed consent is a foundational element of medical ethics and is critical in the deployment of AI systems in healthcare. Amann et al. have emphasized that informed consent represents a patient’s autonomous decision to allow medical intervention, which necessitates full disclosure of the risks, benefits, and the nature of the procedures involved, including AI applications [59]. This transparency is essential for fostering patient autonomy, given that patients must be aware of how AI technologies affect their care and personal data. Radiology-specific ethical challenges in AI governance—such as dataset bias, explainability of image-based AI, and patient consent in clinical imaging—are summarized in Table 3.

In addition to global harmonization efforts, special consideration should be given to regions such as the Middle East, where no comprehensive AI governance frameworks for radiology currently exist. Based on the findings of this review, three recommendations are particularly relevant:

1. Regional Harmonization: establish a unified framework across Middle Eastern countries, modeled after initiatives such as the European Union’s AI Act, to ensure consistency in standards for safety, transparency, and accountability.

2. Capacity Building: develop training programs for radiologists, data scientists, and legal experts in the region to strengthen their expertise in AI ethics, data governance, and regulatory compliance.

3. Contextual Adaptation of Global Models: while borrowing principles from GDPR, HIPAA, and FDA SaMD is valuable, governance models in the Middle East should be adapted to the local healthcare infrastructure, cultural values, and patient rights to ensure sustainable and ethically appropriate AI integration.

By implementing these measures, the Middle East could avoid regulatory fragmentation, foster trust among clinicians and patients, and position itself as a proactive participant in shaping the global dialogue on trustworthy AI in radiology.

Looking ahead, several areas will be critical for the trustworthy and effective integration of AI in radiology. These include the following:

Bridging the gap between ethical theory and clinical practice: ethical frameworks must move beyond abstract principles and be operationalized into daily radiology workflows, ensuring that fairness, transparency, and accountability are applied consistently in clinical decision making.

Developing adaptive regulatory models: current oversight systems are not well suited for continuously learning AI tools. Future governance should include adaptive mechanisms, such as dynamic post-market monitoring, that balance innovation with patient safety.

Strengthening interdisciplinary collaboration: sustainable governance will require active collaboration among radiologists, policymakers, AI developers, ethicists, and legal experts. Establishing international task forces or consortia could help to harmonize standards and accelerate knowledge transfer across regions.

Expanding the use of explainable AI (XAI): future frameworks must integrate XAI requirements, ensuring that radiologists can interpret AI outputs, justify diagnostic decisions, and maintain patient trust.

Building resilience to global health emergencies: the COVID-19 pandemic highlighted the need for agile governance models that are capable of ensuring safe, rapid AI deployment in times of crisis without compromising patient safety.

Addressing regional gaps (e.g., Middle East): dedicated efforts are needed to establish context-specific governance frameworks in regions currently lacking them, adapting global models such as GDPR, HIPAA, and FDA SaMD to local healthcare systems, infrastructure, and cultural contexts.

Recommendations: To ensure trustworthy and effective governance of AI in radiology, several recommendations tailored for specific stakeholders are presented below.

For Medical Practitioners
  - Integrate explainable AI (XAI) into workflows: clinicians should prioritize the adoption of interpretable AI tools (e.g., Grad-CAM, SHAP) to strengthen medico-legal defensibility and patient trust.
  - Enhance training and education: radiologists should receive continuous professional development on AI ethics, liability, and regulatory compliance to prepare for evolving responsibilities.
  - Promote patient-centered governance: emphasize informed consent, transparent data stewardship, and equity in the deployment of AI tools.
  - Engage in governance and policy-making: active participation in hospital boards, professional societies, and regulatory discussions is essential to ensure that clinical perspectives shape AI governance frameworks.
For AI Developers
  - Prioritize dataset diversity and fairness: implement rigorous bias detection and mitigation strategies, particularly for under-represented populations and imaging modalities.
  - Design for explainability and usability: ensure that AI outputs are interpretable for clinicians, balancing technical rigor with clinical applicability.
  - Ensure regulatory alignment: guarantee compliance with GDPR, HIPAA, FDA SaMD, and emerging oversight mechanisms during the development process.
  - Collaborate with clinical experts: engage radiologists throughout the design lifecycle to align algorithms with real-world diagnostic workflows and constraints.
For Policymakers in the Middle East
  - Regional harmonization: develop unified frameworks across Middle Eastern countries, modeled on successful international initiatives, to ensure consistent standards for safety, accountability, and transparency.
  - Capacity building: invest in training for radiologists, legal scholars, and AI specialists to strengthen regional expertise in governance.
  - Contextual adaptation: tailor global models (GDPR, HIPAA, FDA SaMD) to reflect local healthcare infrastructure, cultural values, and patient rights.
For Future Research
  - Empirical evaluation of XAI methods: assess how Grad-CAM, SHAP, and similar approaches impact clinical decision making and medico-legal defensibility in real-world radiology settings.
  - Radiology-specific liability frameworks: explore new medico-legal models for shared responsibility between radiologists and AI systems.
  - Comparative regulatory studies: systematically evaluate outcomes under EU, US, and Canadian frameworks to identify best practices.
  - Bias mitigation strategies: develop protocols for equitable performance across populations and imaging modalities.
  - Middle Eastern context: conduct region-specific studies on regulatory readiness, cultural considerations, and policy development.
  - Dynamic oversight models: investigate adaptive monitoring mechanisms for continuously learning AI systems.

## 4. Conclusions

The integration of artificial intelligence into radiology represents a transformative opportunity to enhance diagnostic accuracy, streamline workflows, and expand access to imaging services. However, its safe and effective deployment depends on governance frameworks that are both technically informed and contextually grounded in radiology practice. This review synthesized findings from 38 studies, highlighting that while ethical principles, legal safeguards, and regulatory instruments exist, they remain fragmented across jurisdictions and are not always tailored to the realities of radiology workflows, such as triage, structured reporting, and population screening.

Recent policy developments, such as the U.S. FDA’s Predetermined Change Control Plan and the EU Artificial Intelligence Act, illustrate evolving efforts to regulate AI responsibly. However, persistent challenges remain in terms of algorithmic bias, explainability, liability, and adaptive oversight. In particular, the Middle East currently lacks comprehensive governance frameworks, underscoring both a pressing challenge and an opportunity to establish regionally tailored regulations.

This review also provides tailored recommendations for different stakeholders: for medical practitioners, focus should be placed on adopting explainable AI, strengthening training, and safeguarding patient rights; for AI developers, the priorities include bias mitigation, regulatory compliance, and collaboration with clinicians; for policymakers (especially in the Middle East), harmonized, context-specific frameworks are needed; and, for future researchers, the agenda includes empirical evaluation of XAI methods, liability models, comparative regulatory studies, and adaptive oversight mechanisms.

By offering guidance to clinicians, developers, policymakers, and researchers alike, this review supports a multi-stakeholder approach to ensuring that innovation in radiology AI proceeds hand in hand with accountability, transparency, and equity.

## Figures and Tables

**Table 1:** Summary of the 38 included studies, indicating source, year, radiology domain, and whether each addressed ethical, legal, and/or regulatory aspects of artificial intelligence in radiology.

Author	Year	Source/Journal	Radiology Domain	Ethical	Legal	Regulatory
Abdullah YI, Schuman JS, Shabsigh R, et al. [1]	2021	Asia-Pacific Journal of Ophthalmology	General healthcare (background)	✔	✘	✘
Pesapane F, Volonté C, Codari M, et al. [2]	2018	Insights into Imaging	General radiology	✔	✘	✔
D’Antonoli TA [3]	2020	Diagnostic and Interventional Radiology	General radiology	✔	✘	✘
Najjar R [4]	2023	Diagnostics	General radiology	✘	✘	✘
Geis JR, Brady AP, Wu CC, et al. [5]	2019	Radiology	General radiology	✔	✘	✘
Singhal A, Neveditsin N, Tanveer H, et al. [6].	2024	JMIR Medical Informatics	General healthcare (background)	✔	✘	✘
Kenny LM, Nevin M, Fitzpatrick K [7]	2021	Journal of Medical Imaging and Radiation Oncology	General radiology	✔	✘	✘
He C, Liu W, Xu J, et al. [8]	2024	iRADIOLOGY	Nuclear medicine	✘	✘	✘
Olorunsogo T, Adenyi AO, Okolo CA, et al. [9].	2024	World Journal of Advanced Engineering Technology and Sciences	General healthcare (background)	✔	✘	✘
Nazer LH, Zatarah R, Waldrip S, et al. [10].	2023	PLOS digital health	General/other	✔	✘	✘
Quazi F [12]	2024	Available at SSRN 4942322	General healthcare (background)	✔	✘	✘
Giansanti D [13]	2022	Healthcare	General radiology	✘	✘	✔
Smith MJ, Bean S [26]	2019	Journal of Medical Imaging and Radiation Sciences	General healthcare (background)	✔	✘	✘
Badawy W, Helal MM, Hashim A, et al. [27].	2025	International Nursing Review	Nuclear medicine	✔	✘	✘
Ejjami R [28]			General healthcare (background)	✔	✘	✘
Koçak B, Ponsiglione A, Stanzione A, et al. [29].	2025	Diagnostic and interventional radiology	Nuclear medicine	✔	✘	✘
Rodriguez-Ruiz A, Lång K, Gubern-Merida A, et al. [15].	2019	Journal of the National Cancer Institute	Mammography/Breast imaging	✘	✘	✘
Wang J, Sourlos N, Heuvelmans M, et al. [30].	2024	Computers in biology and medicine	Chest imaging	✔	✘	✘
McKinney SM, Sieniek M, Godbole V, et al. [31].	2020	Nature	Mammography/Breast imaging	✘	✘	✘
McLennan S, Meyer A, Schreyer K, et al. [32].	2022	PLOS Digital Health	Chest imaging	✘	✘	✘
Goisauf M, Cano Abadía M [33]	2022	Frontiers in Big Data	General radiology	✔	✘	✘
Group CAoRAIW [25]	2019	Canadian Association of Radiologists Journal	General radiology	✔	✔	✘
Currie G, Hawk KE [34]	2021	Seminars in Nuclear Medicine	Nuclear medicine	✔	✔	✘
Ueda D, Kakinuma T, Fujita S, et al. [35].	2024	Japanese Journal of Radiology	General radiology	✔	✘	✘
Harvey HB, Gowda V [36]	2022	Skeletal Radiology	Nuclear medicine	✘	✔	✘
Gerke S, Minssen T, Cohen G [37]	2020	Artificial intelligence in healthcare: Elsevier 2020: 295–336.	General healthcare (background)	✔	✔	✘
Čartolovni A, Tomičić A, Lazić Mosler E [38]	2022	International Journal of Medical Informatics	General healthcare (background)	✔	✔	✘
Union E [22]	2021	COM/2021/206 final	Chest imaging	✘	✘	✔
Voigt P, Von dem Bussche A [39]	2017	A practical guide, 1st ed, Cham: Springer International Publishing	Chest imaging	✘	✘	✔
Price WN, Cohen IG [40]	2019	Nature medicine	General healthcare (background)	✔	✘	✘
Annarumma, M., [18]	2019	Radiology	Chest imaging	✘	✘	✔
FDA U [41]	2019	US Food and Drug Administration	General healthcare (background)	✘	✘	✔
Brown NA, Carey CH, Gerry EI [42]	2021	The Journal of Robotics, Artificial Intelligence & Law	Chest imaging	✘	✔	✔
Administration US FDA [21]	2023	In Administration USFaD, (Ed) 2023.	Chest imaging	✘	✘	✔
Ardila, D [14]	2019	Nat. Med	Chest imaging	✘	✘	✘
Canada H [23]	2019	Health Canada 2019.	General healthcare (background)	✘	✘	✔
Hickman SE, Baxter GC, Gilbert FJ [43]	2021	British Journal of Cancer	Mammography/Breast imaging	✔	✘	✘
Bianchini, E.; Mayer, C. [44]	2022	Artery Res	General healthcare (background)	✘	✘	✔

**Table 2:** Jurisdictional approaches to AI governance in radiology.

Aspect	EU (GDPR + AI Act)	US (HIPAA + FDA SaMD)	Canada (Hybrid)
Legal Scope	GDPR covers all personal health data; AI Act classifies radiology AI as ‘high-risk’	HIPAA limited to ‘covered entities’; FDA regulates SaMD	Risk-based model under Health Canada; aligns with CAR guidance
Operational Requirements	Explicit consent, data minimization, conformity assessment, post-market monitoring	Easier data aggregation, PCCP for adaptive AI, TPLC lifecycle monitoring	Transparency, mandatory human oversight, cross-border alignment
Liability	Strong patient rights; unclear on shared AI liability	Physician-centric with evolving shared liability models	Shared liability framework under development
Cross-Border Compatibility	Strictest; GDPR can conflict with US data practices	Flexible; US-trained models may fail GDPR standards	Serves as a harmonization bridge

**Table 3:** Radiology-specific ethical challenges in AI governance, highlighting key studies that address dataset bias, explainability of image-based AI, and patient consent considerations in clinical imaging.

Author (Year)	Imaging Focus	Ethical Concern	Key Findings
Rodriguez-Ruiz et al. (2019) [15]	Mammography	Bias in datasets	A stand-alone AI system showed subgroup variation: higher recall in dense breast tissue but lower specificity in older women.
McKinney et al. (2020) [31]	Breast cancer screening	Bias in datasets	International AI evaluation revealed ~12% drop in sensitivity in minority populations due to dataset imbalance.
Goisauf & Cano Abadía (2022) [33]	General radiology	Explainability and Consent	Highlighted the ethical implications of opaque AI models and called for dynamic consent to preserve patient autonomy.
Amann et al. (2020) [59]	Healthcare AI (incl. radiology)	Explainability	Stressed that lack of interpretability in AI systems undermines clinician accountability and patient trust.
